# Self-efficacy in exercise behaviour in persons with a diagnosed condition: a systematic evidence map

**DOI:** 10.1136/bmjopen-2025-100029

**Published:** 2026-01-03

**Authors:** Vanessa Bill, Flora Sonsmann, Julian Rafael Rottschäfer, Annika Wilke

**Affiliations:** 1 Institute for Interdisciplinary Dermatological Prevention and Rehabilitation, Osnabrück, Germany; 2 Department of Dermatology, Environmental Medicine and Health Theory, Osnabrück University, Osnabrück, Germany; 3 European Neuroscience Institute Göttingen, Göttingen, Germany

**Keywords:** Exercise, Cardiology, Diabetes & endocrinology, Obesity, PUBLIC HEALTH, Psychometrics

## Abstract

**Abstract:**

**Objectives:**

Self-efficacy is a major factor in enabling individuals to follow behavioural goals. This applies to health behaviours including physical activity and exercise behaviour, a health topic especially important for persons suffering from health conditions. In subjects with already existing conditions, self-efficacy in exercise behaviour is a research field with a high volume of published articles, yet it has never been charted in its entirety. This systematic evidence map (SEM) provides a comprehensive overview of the current state in published empirical research.

**Design:**

Collecting, categorising and visualising the breadth of evidence via SEM following the Methods of Evidence Mapping by Schmucker *et al*.

**Data sources:**

Medline (via PubMed) and PsycINFO (via EbscoHost).

**Eligibility criteria for selecting studies:**

We searched for the terms ‘self-efficacy’ and any of the search terms ‘sport’ and ‘exercise’ in titles and abstracts. We included all empirical research studies published until 2022 that measured self-efficacy in relation to exercise. This SEM includes all studies on humans with a pre-existing condition. We extracted the data points authors, title, year, sample size (N), age groups, pre-existing condition(s), surveyed sport and method of measuring self-efficacy.

**Data extraction and synthesis:**

We extracted the data points from the full text (if available). In addition to a data table, we created a freely accessible evidence map in the form of graphs in this article.

**Results:**

The number of publications grew over time from single publications per year in the 1980s to over 100 per year in the beginning of the 2020s, adding up to 1342 included studies. Most research focuses on middle-aged and older adults. Research covers a wide variety of conditions, with endocrine, nutritional and metabolic diseases (22%) as well as diseases of the circulatory system (19%) being the most common disease groups. Most included studies (71%) do not specify a sport. Most (55%) papers used validated scales to measure self-efficacy, and we discovered 235 individually named scales among them.

**Conclusions:**

This paper offers the first ever comprehensive list of empirical publications on self-efficacy in exercise behaviour in persons with pre-existing conditions in the form of a SEM. The research field was as wide as anticipated concerning total numbers, number of individual scales for measuring self-efficacy, as well as range in diagnosed conditions. Most research focusing on advanced age may be due to many diseases only manifesting later in life, and the lack of specification in types of sport points to the choice of sport being less important than getting enough exercise in general. Future research should examine the strength of evidence and the robustness and comparability of self-efficacy scales as well as underrepresented disease groups for public health considerations.

**Ethics and dissemination:**

Since no primary data was collected, an ethics approval is not required for the presented work. In addition to the result being disseminated via the publication at hand, the data is being shared in detail via the Open Science Framework platform.

**STRENGTHS AND LIMITATIONS OF THIS STUDY**

- A special strength is the systematic approach to charting empirical research on self-efficacy in exercise behaviour in individuals with a pre-existing condition for the first time.
- The systematic evidence map is accessible via the Open Science Framework in a practical and user-friendly format.
- A sensitive search ensured the overview’s comprehensive extent within the set parameters while selection criteria with a high separation power ensured conciseness.
- A limitation of this study is that it does not offer an in-depth analysis of the research topic, thus excluding outcomes and strength of evidence.
- Grey literature is not included.

## Introduction

 The importance of exercise and other physical activity for human health is undisputed and the risks of sedentary behaviour are both commonly known and substantiated by a plethora of empirical evidence.^1^,^3^

This applies to healthy humans in the form of primary prevention,^4^ but also particularly to persons with already existing health conditions in the form of secondary prevention:^5 6^ Besides the universally recommended weekly dose of at least 150 min of moderate-intensity aerobic exercise (or 75 min of vigorous exercise) in addition to two sessions of strength exercise, the WHO guidelines recommend adult cancer survivors, people with hypertension, diabetes or HIV to incorporate functional balance and strength training into their exercise routine.^5^

An active lifestyle is equally important for the secondary prevention of numerous conditions. Following the definition by Frederix *et al*, secondary prevention aims to stop and/or slow down the progress of established (…) disease, to improve functional capacity, to restore quality of life and to reduce the risk of disease recurrence’,^6^ there is plentiful evidence for positive effects of exercise for numerous conditions: for stroke, inactivity is a lifestyle risk factor.^7^ After a stroke, exercise can improve cardiovascular fitness, walking ability and upper-body muscle strength in patients, and help with post-stroke fatigue.^8^ Obese people can benefit from exercise contributing to weight loss, and from an improved cardiorespiratory fitness,^9^ which reduces mortality even when controlling for body mass index (BMI).^10 11^ An active lifestyle can reduce the incidence and improve aetiopathology of heart disease, hypertension, type II diabetes and osteoporosis.^12^,^16^ For a number of cancer types, there is evidence on physical activity reducing not only incidence but also mortality if the cancer already occurred.^17^ For depressive symptoms, recreational physical activity has been shown to be an independent predictive factor^18^ that may reduce the risk of symptom occurrence, as well as improve symptoms in a population with diagnosed depression.^19 20^ The same has been shown for anxiety disorders like panic disorder and agoraphobia,^21^,^23^ although the effects seem to be limited to aerobic exercise.

However, despite these positive effects, a substantial proportion of the human population leads a sedentary lifestyle.^24 25^ For example, in one large cohort study comprising middle-aged US adults, more participants reported daily overall sitting times of >7 hour than <3 hour, with longer sitting times being associated with a significantly higher mortality over 8.5 years.^26^ A similar extent of inactivity and sedentary behaviour is being reported from all over the globe, with comparable escalations of rates of non-communicable diseases and mortality.^27^

The reasons for this discrepancy are complex. The mere knowledge of a risk factor is not enough to change health behaviour, and additional information or fear appeals have little to no effect.^28^ When talking about physical activity, it’s usually trivial to know how to physically get moving, but the difficulty is to actually do it, even when faced with barriers. Barriers to an active lifestyle can be problems of everyday life like time constraints,^29^ cost^30^ or compatibility issues with daily life like child care responsibilities.^31^ People with already existing conditions can also suffer limiting health reasons and symptoms like pain or fatigue.^30 31^ Additionally, there can be psychological barriers within the individual, such as lack of motivation^31^ or failure to implement the necessary intended behaviour, known as intention-behaviour gap.^32^

Researchers in public health have considered different factors that influence this gap and have found some consistently emerging ones. Self-efficacy is one of ‘the most important predictors’ of exercise behaviour.^30^ It is consistently found in empirical research, as shown by systematic reviewing^33^ and influential health behaviour models like the theory of planned behaviour^34^ or the Health Action Process Approach.^35^

Bandura was first to describe the concept and defined self-efficacy as ‘conviction that one can successfully execute the behaviour required to produce the outcomes’,^36^ a necessary cognitive component in bridging the gap between a desire to change in a certain direction, and the action for that change.

Due to the concept’s relevance, there is an expansive body of research about helping people with certain conditions to increase their activity levels, focusing on self-efficacy for exercise behaviour.^37^ The extensive quantity is also complex since numerous empirical studies are dedicated to one individual disease. The studies often apply specifically developed scales for measuring self-efficacy concerning exercise in their exact target group. This is due to the aforementioned limiting health reasons and symptoms like pain or fatigue that may be linked with psychological barriers. One example for such distinct barriers can be found in the Self-efficacy for Physical Activity Scale^33^ that was constructed for women with fibromyalgia and encompasses the item ‘How confident are you that you can walk fast to do exercise over 90 min at least twice a week despite feeling fatigue?’.

This paper aims to provide orientation and improve accessibility to the field of self-efficacy in exercise behaviour by charting this expansive research topic and creating a comprehensive overview. It compiles the published empirical research on self-efficacy concerning exercise behaviour in persons with a pre-existing condition in the practical form of a systematic evidence map (SEM).

## Methods

SEMs are a form of systematic reviewing that allows mapping an extensive research field with a relatively high volume of studies, thus making them the ideal choice for creating an efficient and user-friendly starting point for researchers enquiring about a (new) topic or investigating gaps in a body of empirical research.^38^

This SEM has been preregistered in the Open Science Framework on 11 November 2022.^39^ The SEM follows the Methods of Evidence Mapping by Schmucker *et al*^40^ that consists of the following steps:

1. Definition and prioritisation of the research question.
2. Systematic literature search.
3. Study selection.
4. Data extraction.
5. Reporting the results.

### Step 1: definition and prioritisation of the research question

Step 1 has been described in a protocol published on 2 August 2023.^37^ The research goals were ‘(1) To provide a comprehensive overview of the current state in published empirical research that focuses on self-efficacy concerning exercise behaviour, (2) To identify the currently existing subtopics of self-efficacy in that context, (3) To compile the quantity of research that has been done on the different subtopics and (4) To enable other researchers to systematically justify and derive research gaps in their specific field’.^37^ The specifics of the research question are located in table 1 in the protocol.^37^

### Step 2: systematic literature search

For the search strategy, we followed steps 1–7 of the literature research manual RefHunter V.5.0^41^ to transform our research question into specific search strings. Our goal was a sensitive search, which is also described in the protocol.^37^

We used the resulting search strings in Medline (via PubMed) and PsycINFO (via EbscoHost). In Medline, we took the following search string: ((sport(Title/Abstract)) OR (exercise(Title/Abstract))) AND (self-efficacy(Title/Abstract)). For PsycINFO, we adjusted the search string to the specifics of the database: (AB sport OR AB exercise) AND AB self-efficacy.

Step 2 has been conducted on 1 August 2023.

### Step 3: study selection

Online supplemental file A shows a Preferred Reporting Items for Systematic Reviews and Meta-Analyses flow chart with the study selection process. The overall search returned 6966 results. After deduplication using the systematic literature software Rayyan,^42^ we screened titles and abstracts of 5593 records, using the inclusion/exclusion criteria published in the protocol.^37^ Reviewer VB independently conducted the study selection in Rayyan. Reviewer JRR independently screened a random sample of 561. We calculated the inter-rater agreement of both reviewers using Cohen’s Kappa^43^ at 50, 100, 250 and 500 collaborator decisions and resolved any disagreements through discussion. The interrater agreement was satisfactory from the start (Cohen’s Kappa=0.64) and improved at each step until it reached near perfect agreement (Cohen’s Kappa=0.96).

We excluded 4198 results based on title and abstract. During the full-text screening, we further eliminated 92 studies due to exclusion criteria only becoming apparent in the full text (reasons for exclusion are listed in online supplemental file A). Furthermore, we included 39 studies from our twin papers’ study pool. In this SEM, we focus on secondary prevention, so only persons with diagnosed condition(s) were included, while in the twin paper, we focus on primary prevention, so only persons without any diagnosed conditions were included. For that paper, in some cases, exclusion criteria also only became apparent after the abstract screening when seeing the full text. In 39 cases, a supposedly healthy sample turned out to have diagnosed condition(s), so we took the opportunity to not only exclude those studies from the primary prevention SEM during the full-text screening, but to also retroactively include them into this SEM.

Step 3 has been started on 2 August 2023 and finished on 12 October 2023.

### Step 4: data extraction

Reviewer VB exported the included studies into Microsoft Excel (V.2024) and extracted the following data points from the full text (if available, else from the abstract) of the remaining 1342 studies: authors, title, year, sample size, age groups, pre-existing condition(s), surveyed sport, method of measuring self-efficacy.

Step 4 lasted from 1 November 2023 to 20 December 2023.

### Step 5: reporting the results

The authors, title and year were automatically exported by the systematic literature software Rayyan. For age groups, pre-existing condition(s), surveyed sport and method of measuring self-efficacy (validated scale^44 45^ or ad hoc scale), we first copy-pasted the relevant text parts and then added a categorisation column, if applicable, for transparency and traceability.

As per protocol, we categorised the age groups based on the Provisional Guidelines on Standard International Age Classifications of the WHO (1982): 1–14, 15–24, 25–44, 45–64, 65+years of age^46^ and marked all applicable age groups. We defined categorisation rules and added them in online supplemental file B.

We decided to add the following information columns in the table:

Digital object identifier or PubMed ID, URL and categorisation for pre-existing condition(s). There was no categorisation for pre-existing conditions planned. After seeing how differently they were named (eg,: obesity vs BMI >30), we decided it would facilitate finding the correct accumulation of listings to add the corresponding letter according to ICD-10 categories (categorisation rules see online supplemental file A). Additionally, if information is missing due to the full text being unavailable to the authors, we marked ‘no full text’ in an additional column.

Step 5 started on 16 January 2024 and was completed on 31 January 2025.

Throughout the work on this SEM, we decided to make the following changes to the protocol:^37^ We exported the studies directly from Rayyan into Excel and extracted the data directly from each original study into Excel due to technical issues with the systematic review programme we originally planned to use (SRDR+^47^). This did not have any negative impact on the research goal or any part of the results. We added the aforementioned information columns. We changed the figure depicting pre-existing conditions from a pie chart to a bar chart for better readability. This had a positive impact on the usefulness and accessibility of the results we present in the SEM.

Since this SEM uses exclusively secondary data, it required no ethics approval.

## Results

Following the protocol, we present our results and depict a selection of data in charts.

**Figure 1:**
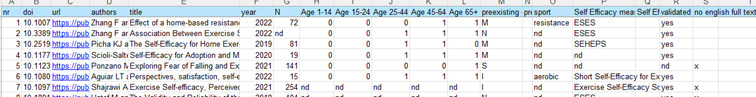
Overview of the extracted data in Excel of the systematic evidence map. Screenshot of a Microsoft Excel file containing the extracted data points for seven studies.

We compiled the extracted data in a Microsoft Excel spreadsheet that we uploaded at https://osf.io/t5g3e.^48^ We included a screenshot for a quick visual impression in figure 1. Subgroups of interest can be displayed in Microsoft Excel by different methods. One of the easiest is using the ‘sort by’ function in the toolbar or the ‘find & replace’ dialogue box to find the relevant subgroups. By using the ‘filter’ function, it’s possible to display solely the subgroups of choice.

**Figure 2:**
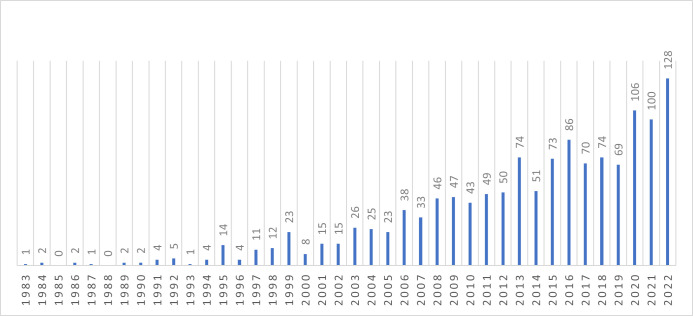
Publications on self-efficacy and exercise behaviour per year. Vertical bar chart. The horizontal baseline shows the publication years, and the individual bars show the number of publications in the respective year.

For the paper at hand, we visualised the following selection of data:

The first paper on self-efficacy and exercise behaviour in persons with a diagnosed condition was published in 1983. The growing number of papers per year is shown in a bar chart (figure 2). While in the 1980s the publications were in the single digits, 1995 marks the first year 14 articles were published. Despite fluctuations, the number of publications generally continued to grow. Since 2020, the numbers of yearly publications have been in the triple digits.

**Figure 3:**
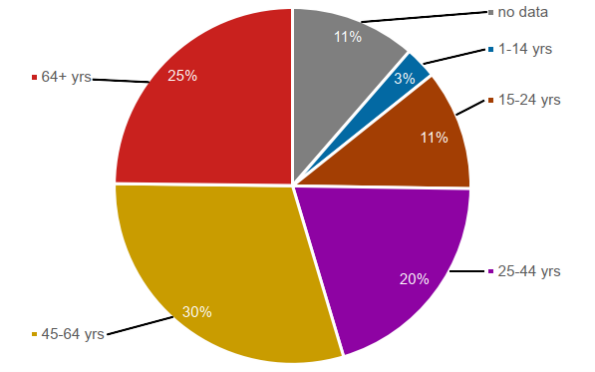
Ratio of represented age groups according to the categorisation of the WHO (1982). Pie chart. Five of the shares show the percentage of age groups represented in the publications, and the sixth share shows the percentage of publications with no data on age groups.

The papers addressed different age groups. For the represented age groups according to the categorisation by the WHO,^46^ we created a pie chart. A selection of multiple age groups in one article was possible. For example, if the ages of a sample in a paper ranged from 20 to 30, we chose the age groups 3 (15–24) and 4 (25–44). A complete list of categorisation rules can be found in online supplemental file A. As figure 3 shows, most publications focus on adults, mainly middle-aged persons or elders, with around 75% of all included papers belonging to age groups 4–6 (25 years and older). The largest age group is age group 5, with 30% of the publications including the age group of 45–64 years.

**Figure 4:**
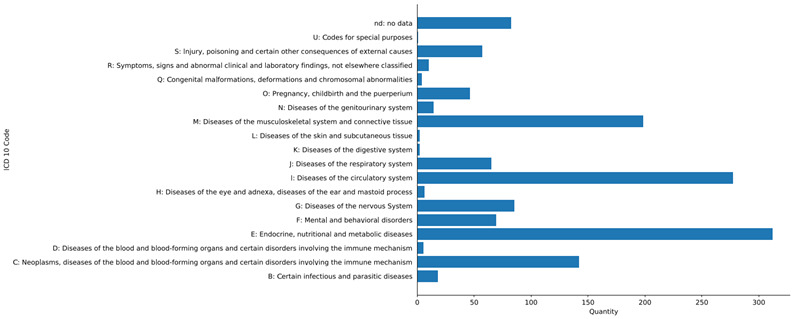
Reported pre-existing conditions using ICD-10 code letters. Horizontal bar chart. The individual bars show the quantity of publications, each bar representing a disease group from the ICD-10 or ‘no data’.

In terms of pre-existing conditions, the studies reported a wide range which we visualised according to the ICD-10 in a bar chart (see figure 4).

The disease groups E (endocrine, nutritional and metabolic diseases) and I (diseases of the circulatory system) are distinctly the largest with 22% and 19%, respectively. Two more condition groups stand out, namely M (diseases of the musculoskeletal system and connective tissue) at 14% and C (neoplasms, diseases of the blood and blood-forming organs and certain disorders involving the immune mechanism) at 11%. The portions of all other disease groups each remain in the single digit percentages.

**Figure 5:**
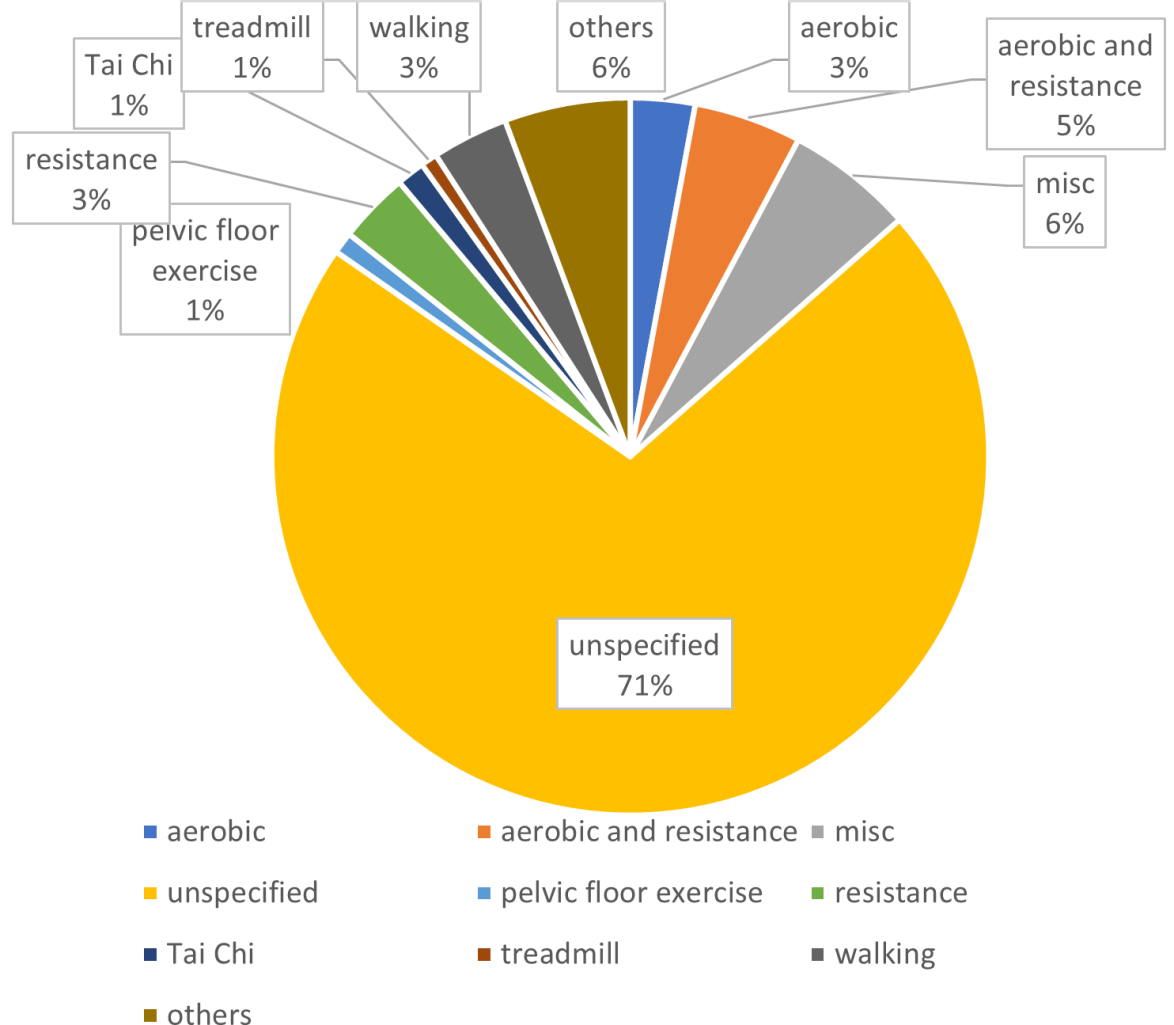
Ratio of sports included in >=10 publications. Pie chart. Each share shows the percentage of specific sports represented in the publications, with one share representing the percentage of publications with unspecified sports.

Since this SEM focuses on self-efficacy in the context of exercise behaviour, the recorded types of sport were of interest, which showed a considerable range (see figure 5). Due to a high number of sports that were mentioned in just a few publications, we decided to only include sports by name if they were included in at least 10 articles. Rarely mentioned sports (found by ‘others’ in figure 5) are added in table 1 below.

**Table 1:** Count of sports included in <10 publications

Sport	Count
Aquatic	4
Baduanjin	2
Bicycle	8
Boccia	1
Bouldering	1
Boxing	1
Breathing exercise	1
Climbing	1
Dance	7
Exergames	5
Golf	1
HIIT	4
Kyusho Jitsu	1
Lactacid anaerobic training	1
Powerlifting	1
Qi	1
Qi Gong	5
Rope skipping	1
Running	4
Schroth	1
Soccer	1
Stretching	4
Swimming	2
Swiss ball	1
Triathlon	1
Wheelchair sports	6
Yoga	9
Zumba	1

When focusing on the instruments used to measure self-efficacy, we determined that 731 articles (54.6%) used validated scales as defined in online supplemental file B, among which we discovered 235 individually named scales. 123 (9.2%) used ad hoc scales. In 485 (36.2%) articles, the validation status was unclear or unspecified. We listed the individually named scales in online supplemental file C. As expected, we found and listed many subgroup-specific scales like the Arthritis Self-Efficacy Scale or the Diabetes Management Self-Efficacy Scale. Nevertheless, more general scales were also used, for example, the Exercise Self-Efficacy Scale (ESE/ESES) that has no specific target group, or indeed the General Self-Efficacy Scale that has neither a target group nor does it even focus on exercise or sport.

## Discussion

This paper constitutes—to the best of our knowledge—the first ever compilation of empirical publications on self-efficacy in exercise behaviour in persons with pre-existing conditions in the form of a SEM. It offers a comprehensive list of empirical papers on this topic. Its result section gives a synopsis of the research field, and the actual SEM allows a deeper dive into certain subsets of the expansive research field. Viewing the literature on the topic, it quickly becomes clear that a synthesis is difficult to achieve due to its complexity. It branches out into situation-specific and target-group-specific sub-topics. This empirical research has never been charted in its entirety.

We found that with its 1342 included publications, the research field was as expansive as anticipated. With a focus on age groups, it was also unsurprising that the majority of publications focused on the second half of life since a number of diseases emerge with age.^49 50^ For example, the pathogenesis of atherosclerosis takes until the age of mid-20s to cause irreversible changes and noticeable symptoms do not emerge until middle age.^51 52^ Similarly, COPD prevalence increases with age^53^ and age is the greatest risk factor for developing cancer.^54^ Because of this, effects of self-efficacy and habits influencing exercise behaviour in these vulnerable populations are highly relevant.

The representation of disease groups was relatively uneven, which may be attributed to differing research interest due to prevalence, public awareness, funding, healthcare costs, lethality and differing pertinence in certain countries and in certain socioeconomic groups.^55^ The reasons might range from rightful difference of interest over unfortunate but understandable reasons like economical possibilities to unsubstantiated neglect. Rightful difference of interest would be different groups having different problems, like more weight-related research in a country with high obesity rates than in a country with low obesity rates. Neglected tropical diseases are an example of underrepresentation due to economic barriers.^56^ Musculoskeletal disorders and skin diseases may be surprising examples of unsubstantiated neglect^57 58^ since they are burdensome diseases to suffer from, costly to the public, and often preventable and/or treatable. With a focus on self-efficacy and exercise behaviour, other barriers may prevent different persons from different health behaviours. Self-efficacy may not even emerge as a topic if cost or accessibility is a prohibitive factor.^59^

In surveyed sports, it became apparent that most of the research does not focus on a single sport, but either includes a variety of sports, or a wide type of sport (eg, aerobic sports, which includes all sports that train the cardiovascular system), or does not even specify the type of sport at all. This is often unsurprising, since the specific choice of sport is not always relevant: if the goal is a cardiovascular effect, like for obesity,^60^ the individual will reap the same benefits from swimming as they do from hiking. Additionally, picking an exercise the individual enjoys (even if it may not be the ideal choice for their physical condition) naturally helps motivation and, therefore, adherence.^61^ A high adherence to the point of exercise behaviour becoming a habit is intertwined with self-efficacy and reliably predicts future behaviour.^62^

This SEM offers some distinct strengths. In concordance with the research goal, it succeeded in gathering the vast number of empirical studies on our research topic in a handy overview. The sensitive search ensured the overview’s comprehensive extent within the set parameters. As already mentioned in the method section, our systematic approach and the choice of systematic evidence mapping, as well as the decision to separate the topics of primary prevention and secondary prevention, allowed us to be concise and yet comprehensive. We achieved a near-perfect inter-rater agreement and therefore have good reason to trust the sharpness of separation.

When compiling a list of all validated scales, there were also some limitations to our literature review and the resulting SEM: some scales measure exercise self-efficacy and a different dimension, but the scale’s name does not indicate it. For example, the Diabetes Management Self-Efficacy did not seem relevant to self-efficacy in exercise behaviour, but it consists of five subscales, one of which measures exercise self-efficacy.^63^ Because of that, we may have wrongly excluded relevant self-efficacy scales. When we compiled our list of validated scales, we also realised that names and abbreviations are not standardised. For example, the ‘ESE’ scale is the abbreviation for ‘exercise self-efficacy’ as well as ‘emotional self-efficacy’ scale.^64^ Conversely, the Osteoporosis self-efficacy scale is sometimes abbreviated as OSES, sometimes as OSE.^65 66^ When speaking about validated scales and ad hoc scales, we also have to note that we have not verified whether each scale’ validation process followed best practice. Furthermore, we included ‘patient’ in the inclusion criteria for the abstract screening on the assumption that this label is limited to persons with a pre-existing condition. This turned out not to always be the case, as became apparent in the full text. In very few cases, the focus group was patients of a preventive care programme, so they did not necessarily have any pre-existing conditions.^67^ In some cases, the distinction between primary prevention (persons without a condition) and secondary prevention (persons with a condition) is not visible in the abstract. Especially in overweight populations, sometimes the target group includes overweight persons but also includes obese persons with a BMI of 30 or more.^68^ It’s also important to note that the studies’ outcomes were out of scope for this publication. Therefore, no information about the effect of self-efficacy on any selected outcome can be gathered directly from the SEM, which would require any interested party to gather them straight from the papers that are listed for their group of interest in this SEM. Additionally, assessment of strength of evidence and grey literature was not included to limit the scope, which is not unusual in such expansive research fields as self-efficacy in exercise behaviour.^38^

In future research, we propose expanding into this direction as well as updating with new studies published in 2023 and later. A review that assesses the robustness of self-efficacy scales and the effects of heterogeneity of wording (as discussed above) would be relevant. A further research direction pertains to how different target groups understand certain phrasing, namely whether self-efficacy statements are usually interpreted as questions of willpower and commitment, or of external barriers like time constraints. It would also help future research to create a nomothetical network of different aspects of self-efficacy in exercise behaviour, to identify the common denominator(s) and to assess the comparability of these specific aspects. We would also propose closing the research gaps we outlined: for public health considerations, it would be important to identify underrepresented disease groups (as discussed above), to which the variation in the represented disease groups in this SEM is a good start. Concerning different sports, we would welcome more research on specific sports and their differences, for example contrasting different moderate intensity sports versus vigorous intensity sports according to the WHO classification.^5^ Also, comparing gender differences (or socioeconomic differences) in certain sports, for example their acceptability, popularity and visibility, which could affect self-efficacy and thus success rates in adapting exercise habits.

Future research should also focus on the relationship between self-efficacy and physical activity. How does a bout of physical activity affect self-efficacy and how long does the influence last? Are there factors that facilitate the stability of any causal effects?

With the existing abundance of health problems that are caused by sedentary behaviour and/or that can be mitigated by physical activity, it is of utmost importance for public health research to find out how to empower people to lead an active lifestyle, to which self-efficacy is one of the bigger influence factors. In conclusion, this SEM constitutes a user-friendly tool for researchers who are approaching a research project with relevance to self-efficacy in exercise behaviour. Starting with this paper and the mapped articles to familiarise themselves with the general research field will save time and effort, allowing an easier and more efficient entry into their own, more specific, topics of interest.

## Supplementary material

10.1136/bmjopen-2025-100029 online supplemental file 1

10.1136/bmjopen-2025-100029 online supplemental file 2

10.1136/bmjopen-2025-100029 online supplemental file 3

## Data Availability

Data are available in a public, open access repository.

## References

[1] Warburton DER, Bredin SSD (2017). Health benefits of physical activity: a systematic review of current systematic reviews. Curr Opin Cardiol.

[2] Malm C, Jakobsson J, Isaksson A (2019). Physical Activity and Sports-Real Health Benefits: A Review with Insight into the Public Health of Sweden. *Sports (Basel*).

[3] Posadzki P, Pieper D, Bajpai R (2020). Exercise/physical activity and health outcomes: an overview of Cochrane systematic reviews. BMC Public Health.

[4] AIHW 8.1 Prevention for a healthier future (Feature)(Chapter 8 – Preventing and treating ill health; Australia's health 2014).

[5] Bull FC, Al-Ansari SS, Biddle S (2020). World Health Organization 2020 guidelines on physical activity and sedentary behaviour. Br J Sports Med.

[6] Frederix I, Dendale P, Schmid J-P (2017). Who needs secondary prevention?. Eur J Prev Cardiol.

[7] Johnson W, Onuma O, Owolabi M (2016). Stroke: a global response is needed. Bull World Health Organ.

[8] Billinger SA, Arena R, Bernhardt J (2014). Physical activity and exercise recommendations for stroke survivors: a statement for healthcare professionals from the American Heart Association/American Stroke Association. Stroke.

[9] Jakicic JM, Otto AD (2006). Treatment and prevention of obesity: what is the role of exercise?. Nutr Rev.

[10] Farrell SW, Braun L, Barlow CE (2002). The relation of body mass index, cardiorespiratory fitness, and all-cause mortality in women. Obes Res.

[11] Wei M, Kampert JB, Barlow CE (1999). Relationship between low cardiorespiratory fitness and mortality in normal-weight, overweight, and obese men. JAMA.

[12] American College of Sports Medicine (1993). Physical Activity, Physical Fitness, and Hypertension. Med Sci Sports Exerc.

[13] Helmrich SP, Ragland DR, Leung RW (1991). Physical activity and reduced occurrence of non-insulin-dependent diabetes mellitus. N Engl J Med.

[14] Leon AS, Connett J, Jacobs DR (1987). Leisure-Time Physical Activity Levels and Risk of Coronary Heart Disease and Death. *JAMA*.

[15] Murray TM, Ste-Marie LG (1996). Prevention and management of osteoporosis: consensus statements from the Scientific Advisory Board of the Osteoporosis Society of Canada. 7. Fluoride therapy for osteoporosis. *CMAJ*.

[16] Pelliccia A, Sharma S, Gati S (2020). ESC Guidelines on sports cardiology and exercise in patients with cardiovascular disease. Eur Heart J.

[17] Rezende LFM de, Sá TH de, Markozannes G (2018). Physical activity and cancer: an umbrella review of the literature including 22 major anatomical sites and 770 000 cancer cases. Br J Sports Med.

[18] Farmer ME, Locke BZ, Mościcki EK (1988). Physical activity and depressive symptoms: the NHANES I Epidemiologic Follow-up Study. Am J Epidemiol.

[19] Martinsen EW, Hoffart A, Solberg O (1989). Comparing aerobic with nonaerobic forms of exercise in the treatment of clinical depression: a randomized trial. Compr Psychiatry.

[20] Heissel A, Heinen D, Brokmeier LL (2023). Exercise as medicine for depressive symptoms? A systematic review and meta-analysis with meta-regression. Br J Sports Med.

[21] Martinsen EW, Hoffart A, Solberg ØY (1989). Aerobic and non‐aerobic forms of exercise in the treatment of anxiety disorders. Stress Med.

[22] Raglin JS (1990). Exercise and mental health. Beneficial and detrimental effects. Sports Med.

[23] Singh B, Olds T, Curtis R (2023). Effectiveness of physical activity interventions for improving depression, anxiety and distress: an overview of systematic reviews. Br J Sports Med.

[24] Jones DA, Ainsworth BE, Croft JB (1998). Moderate leisure-time physical activity: who is meeting the public health recommendations? A national cross-sectional study. Arch Fam Med.

[25] Sabe M, Chen C, Sentissi O (2022). Thirty years of research on physical activity, mental health, and wellbeing: A scientometric analysis of hotspots and trends. Front Public Health.

[26] Matthews CE, George SM, Moore SC (2012). Amount of time spent in sedentary behaviors and cause-specific mortality in US adults. Am J Clin Nutr.

[27] Lee I-M, Shiroma EJ, Lobelo F (2012). Effect of physical inactivity on major non-communicable diseases worldwide: an analysis of burden of disease and life expectancy. Lancet.

[28] Kelly MP, Barker M (2016). Why is changing health-related behaviour so difficult?. Public Health (Fairfax).

[29] Thapaliya R, Leshner G, Sharma Ghimire P (2022). An extension of the extended parallel process model to promote heart-healthy exercise behavior: An experimental study. Health Promot Perspect.

[30] Kasser SL, Kosma M (2012). Health beliefs and physical activity behavior in adults with multiple sclerosis. Disabil Health J.

[31] Booth ML, Bauman A, Owen N (1997). Physical Activity Preferences, Preferred Sources of Assistance, and Perceived Barriers to Increased Activity among Physically Inactive Australians. Prev Med.

[32] Rhodes RE, Cox A, Sayar R (2022). What Predicts the Physical Activity Intention-Behavior Gap? A Systematic Review. Ann Behav Med.

[33] López-Roig S, Pastor-Mira M-Á, Núñez R (2021). Assessing Self-Efficacy for Physical Activity and Walking Exercise in Women with Fibromyalgia. Pain Manag Nurs.

[34] Ajzen I (1991). The theory of planned behavior. Organ Behav Hum Decis Process.

[35] Schwarzer R, Renner B (2000). Social-cognitive predictors of health behavior: action self-efficacy and coping self-efficacy. *Health Psychol*.

[36] Bandura A (1978). Self-efficacy: Toward a unifying theory of behavioral change. Advanc Behav Res Ther.

[37] Bill V, Wilke A, Sonsmann F (2023). What is the current state of research concerning self-efficacy in exercise behaviour? Protocol for two systematic evidence maps. BMJ Open.

[38] Miake-Lye IM, Hempel S, Shanman R (2016). What is an evidence map? A systematic review of published evidence maps and their definitions, methods, and products. Syst Rev.

[39] OSF Registries Self-efficacy in exercise behaviour in secondary prevention: a systematic evidence map 2024.

[40] Schmucker C, Motschall E, Antes G (2013). Methoden des Evidence Mappings. *Bundesgesundheitsbl*.

[41] 10 Schritte zur systematischen Literaturrecherche – RefHunter 2024.

[42] Ouzzani M, Hammady H, Fedorowicz Z (2016). Rayyan-a web and mobile app for systematic reviews. Syst Rev.

[43] McHugh ML (2012). Interrater reliability: the kappa statistic. Biochem Med (Zagreb).

[44] Boateng GO, Neilands TB, Frongillo EA (2018). Best Practices for Developing and Validating Scales for Health, Social, and Behavioral Research: A Primer. Front Public Health.

[45] Bland JM, Altman DG (2002). Statistics Notes: Validating scales and indexes. BMJ.

[46] Department of International Economic And Social Affairs- Statistical Office (1982). Provisional guidelines on standard international age classifications.

[47] (2024). SRDR+ 2024.

[48] Bill V, Sonsmann F, Rottschäfer J (2025). Data from: Self efficacy in exercise behaviour in persons with a diagnosed condition: A systematic evidence map. Open Science Framework.

[49] Determinanten der Gesundheit 2024.

[50] Statista (2024). Gesundheitszustand - Verbreitung chronischer Krankheiten unter Männern in Deutschland nach Alter 2021.

[51] Jebari-Benslaiman S, Galicia-García U, Larrea-Sebal A (2022). Pathophysiology of Atherosclerosis. Int J Mol Sci.

[52] Man JJ, Beckman JA, Jaffe IZ (2020). Sex as a Biological Variable in Atherosclerosis. Circ Res.

[53] Agarwal AK, Raja A, Brown BD (2023). Chronic obstructive pulmonary disease.

[54] Newell GR, Spitz MR, Sider JG (1989). Cancer and age. Semin Oncol.

[55] Timmis A, Vardas P, Townsend N (2022). European Society of Cardiology: cardiovascular disease statistics 2021. Eur Heart J.

[56] Aerts C, Sunyoto T, Tediosi F (2017). Are public-private partnerships the solution to tackle neglected tropical diseases? A systematic review of the literature. *Health Policy*.

[57] Perruccio AV, Yip C, Badley EM (2017). Musculoskeletal Disorders: A Neglected Group at Public Health and Epidemiology Meetings?. Am J Public Health.

[58] Khatami A, San Sebastian M (2009). Skin disease: a neglected public health problem. Dermatol Clin.

[59] Chen MH, DeLuca J, Sandroff BM (2022). Aquatic exercise for persons with MS: Patient-reported preferences, obstacles and recommendations. Mult Scler Relat Disord.

[60] Annesi JJ, Gorjala S (2010). Changes in Theory-Based Psychological Factors Predict Weight Loss in Women with Class III Obesity Initiating Supported Exercise. J Obes.

[61] Collado-Mateo D, Lavín-Pérez AM, Peñacoba C (2021). Key Factors Associated with Adherence to Physical Exercise in Patients with Chronic Diseases and Older Adults: An Umbrella Review. Int J Environ Res Public Health.

[62] Grasaas E, Sandbakk Ø (2024). Adherence to physical activity recommendations and associations with self-efficacy among Norwegian adolescents: trends from 2017 to 2021. Front Public Health.

[63] D’Souza MS, Karkada SN, Parahoo K (2017). Self-efficacy and self-care behaviours among adults with type 2 diabetes. Appl Nurs Res.

[64] Valois RF, Umstattd MR, Zullig KJ (2008). Physical activity behaviors and emotional self-efficacy: is there a relationship for adolescents?. J Sch Health.

[65] Elliott JO, Jacobson MP, Seals BF (2006). Self-efficacy, knowledge, health beliefs, quality of life, and stigma in relation to osteoprotective behaviors in epilepsy. Epilepsy Behav.

[66] Solimeo SL, Nguyen V-TT, Edmonds SW (2019). Sex differences in osteoporosis self-efficacy among community-residing older adults presenting for DXA. *Osteoporos Int*.

[67] Ylitalo KR, Cox W, Gutierrez M (2020). A Prescription for Wellness: Exercise Referrals at a Federally Qualified Health Center. *J Prim Care Community Health*.

[68] Gallagher KI, Jakicic JM, Napolitano MA (2006). Psychosocial factors related to physical activity and weight loss in overweight women. Med Sci Sports Exerc.

