# Supplementary material for: Self-efficacy in exercise behaviour in persons with a diagnosed condition: a systematic evidence map
Source: BMJ Open. 2026 Jan 3;16(1):e100029. doi: 10.1136/bmjopen-2025-100029 (PMC12766792; doi:10.1136/bmjopen-2025-100029)
Supplement: online supplemental file 1 [file bmjopen-16-1-s001.docx]

**Identification of studies via databases and registers**

**Primary prevention**

**Secondary prevention**

Records removed *before screening*:

Duplicate records removed (n=1373)

Records marked as ineligible by automation tools (n=0)

Records removed for other reasons (n=0)

Records identified from*:

Databases (n=2)

Medline via PubMed: (n=3831)

PsycINFO via EbscoHost:

(n=3135)

Registers (n=0)

**Identification**

Records included in primary prevention**

(n=1567)

Records screened

(n=5593)

Records excluded**

(n=4198)

Reports sought for retrieval

(n=1395)

Reports not retrieved

(n=0)

**Screening**

Records included in primary prevention**

No preexisting condition (n=20)

92 Reports excluded:

No preexisting condition (n=20)

No scale was used (n=33)

Not empirical research (n=30)

Self-efficacy not toward exercise behaviour (n=5)

Article is retracted (n=3)

Anthology instead of article (n=1)

Reports assessed for eligibility

(n=1395)

Reports from primary prevention

(n=39)

Studies included in review

(n=1342)

**Included**

*Consider, if feasible to do so, reporting the number of records identified from each database or register searched (rather than the total number across all databases/registers).

**If automation tools were used, indicate how many records were excluded by a human and how many were excluded by automation tools: All records were excluded by a human. An automation tool provided by the systematic literature review software Rayyan was used for calculating similarity ratings for quicker screening.

*From:*  Page MJ, McKenzie JE, Bossuyt PM, Boutron I, Hoffmann TC, Mulrow CD, et al. The PRISMA 2020 statement: an updated guideline for reporting systematic reviews. BMJ 2021;372:n71. doi: 10.1136/bmj.n71

For more information, visit: <http://www.prisma-statement.org/>
