# Supplementary material for: Self-efficacy in exercise behaviour in persons with a diagnosed condition: a systematic evidence map
Source: BMJ Open. 2026 Jan 3;16(1):e100029. doi: 10.1136/bmjopen-2025-100029 (PMC12766792; doi:10.1136/bmjopen-2025-100029)
Supplement: online supplemental file 2 [file bmjopen-16-1-s002.docx]

**Supplementary file b: Complete list of categorisation rules**

**Categories of age:**

If no age range, but a mean and standard deviation was provided, we included the mean age category and one standard deviation to either side. If only the mean was mentioned, solely the age category including this mean was included.

The listed terms were categorised as follows:

Childhood, elementary school children: 1-14

Schoolchildren: 1-14, 15-24.

Adolescents, youth: 1-14,15-24

College students: 15-24, 25-44

Young adults: 15-24, 25-44

Adults: all except 1-14

Middle-aged: 45-64

Older, elderly, elders: 65+

**Categories of scale validation status:**

The scale was deemed validated if either the scale was named, the validation study of said scale was linked or the authors explicitly stated that the scale was validated. If a scale had an abbreviation, we put that as the name in a separate column. If several scales were used, we added those scales in a second column.

**Categorisation of pre-existing conditions or diseases:**

The pre-existing conditions were listed with the corresponding letter according to the ICD-10. All conditions related to pregnancy and postpartum were coded “O”. For hemodialysis patients, we assumed an underlying kidney disease and coded “N”. Studies about patients with diseases of the musculoskeletal systems were coded “M”, including implied conditions, e.g. “knee replacement” or “spinal disc surgery”.

If a second pre-existing condition with a different corresponding letter was listed (e.g. COPD and diabetes), it was listed in a second column.

If no specific condition was listed, e.g. “with disabilities” or “chronic disease”, we put “nd”.

**Categorisation of sports:**

If one specific sport was named, we left it unchanged.

If different specific sports from different categories were named, we categorised as misc for miscellaneous.

We found that a lot of studies focused on types of training, namely strength training or aerobic training of the cardiovascular system. Because of that, we decided to add those as categories:

Aerobic: cardiovascular exercise, endurance. We also chose this category if several specific sports from this category were named, e.g. “biking, treadmill running or hiking”.

Resistance: Aerobic and resistance if both. Including: Functional exercise

HIIT if specifically mentioned.

Nd: step count, accelerometer, sports, exercise, gym membership. Or no data due to unspecified sport.
