# Supplementary material for: Self-efficacy in exercise behaviour in persons with a diagnosed condition: a systematic evidence map
Source: BMJ Open. 2026 Jan 3;16(1):e100029. doi: 10.1136/bmjopen-2025-100029 (PMC12766792; doi:10.1136/bmjopen-2025-100029)
Supplement: Supplementary file 4 [file bmjopen-2025-100029.reviewer_comments.pdf]

PEER REVIEW HISTORY

BMJ Open publishes all reviews undertaken for accepted manuscripts. Reviewers are asked to complete a checklist review form and are provided with free text boxes to elaborate on their assessment. These free text comments are reproduced below.

ARTICLE DETAILS

Title (Provisional)

Self-efficacy in exercise behaviour in persons with a diagnosed condition: A systematic evidence map

Authors

Bill, Vanessa; Sonsmann, Flora; Rottschäfer, Julian Rafael; Wilke, Annika

---

VERSION 1 - REVIEW

**Reviewer**	**1**
**Name**	**Bamonti, Patricia M**
**Affiliation**	**VA Boston Healthcare System**
**Date**	**05-Jun-2025**
**COI**	**None**

Thank you for the opportunity to review the manuscript titled “Self-efficacy in exercise behaviour in persons with a diagnosed condition: A systematic evidence map.” This systematic evidence map aimed to provide an overview of the research evidence of self-efficacy of exercise behavior in participants with pre-existing health conditions. The results present number of publications over time, break-down of disease conditions, type of sport, and list of measurement instruments and whether validated (or not). The main criticism of the evidence map is lack of attention to the effect of exercise self-efficacy on selected outcomes. The Introduction sets up exercise self-efficacy as an important construct for exercise behavior, but the systematic evidence map does not address effects. The data collected is limited to characteristics of the #, type of sport, and instrument, but these data provide limited information in terms of gaps and directions to the readers. Below is section-specific feedback:

Introduction:

- The Introduction can be tightened by reducing amount of text devoted to the health benefits of physical activity and exercise and more quickly moving to the focus on exercise self-efficacy.

- The authors discuss several constructs relevant to exercise behavior (p. 5, lines 27-34). I think the authors would benefit from introducing self-efficacy in the context of existing behavior change theories and prior literature without delineating other constructs related to exercise behavior. The focus of the evidence map is on self-efficacy, so no need to introduce other constructs to the reader. Tell us why you are focused on self-efficacy for exercise.

- Minor. “Self-efficacy in exercise behavior” should be “self-efficacy for exercise behavior.”

Results:

- Commonly, systematic evidence map provides a figure of the map of the evidence. The authors do not provide an evidence map. The main limitation is that the authors do not map the evidence of the effects of exercise self-efficacy for key outcomes.

Discussion:

- Overall, the Discussion sufficiently covers the findings, but is challenged by the lack of relevant gaps and research directions provided by this review. The utility of the evidence map for researchers is limited.

**Reviewer**	**2**
**Name**	**Rolid, Katrine**
**Affiliation**	**Oslo University Hospital, Dep of Cardiology**
**Date**	**05-Jul-2025**
**COI**	**None**

Thank you for the opportunity to review this interesting paper about self-efficacy in exercise behavior. The paper is well written and will be an informative guide for future researchers in the field. I have some comments to some of the references which is of older date , especially the papers regarding mental health (references 22- 26). The authors could consider to include references of newer date for example Heissel A, et al. Exercise as medicine for depressive symptoms? A systematic review and meta-analysis with meta-regression British Journal of Sports Medicine 2023;57:1049-1057 , doi:10.1136/bjsports-2022-1062 or Singh B et al . Effectiveness of physical activity interventions for improving depression, anxiety and distress: an overview of systematic reviews. Br J Sports Med 2023;57:1203–9. doi:10.1136/bjsports-2022-106195

VERSION 1 - AUTHOR RESPONSE

Reviewer: 1
Dr. Patricia M Bamonti, VA Boston Healthcare System
Comments to the Author:
Thank you for the opportunity to review the manuscript titled “Self-efficacy in exercise behaviour in persons with a diagnosed condition: A systematic evidence map.” This systematic evidence map

aimed to provide an overview of the research evidence of self-efficacy of exercise behavior in participants with pre-existing health conditions. The results present number of publications over time, break-down of disease conditions, type of sport, and list of measurement instruments and whether validated (or not). The main criticism of the evidence map is lack of attention to the effect of exercise self-efficacy on selected outcomes. The Introduction sets up exercise self-efficacy as an important construct for exercise behavior, but the systematic evidence map does not address effects. The data collected is limited to characteristics of the #, type of sport, and instrument, but these data provide limited information in terms of gaps and directions to the readers.

ANSWER: Thank you for the appreciation of our research topic and for your valuable time in reviewing our manuscript.

We agree that effects are highly relevant in the context of self-efficacy of exercise behaviour. We addressed this important point in the discussion: "Future research should also focus on the relationship between self-efficacy and physical activity. How does a bout of physical activity affect self-efficacy and how long does the influence last? Are there factors that facilitate the stability of any causal effects?"

However, these research questions were not within the scope of this systematic evidence map as per protocol published in 2023 (Link: https://bmjopen.bmj.com/content/13/8/e070359). Our main goal was to provide a comprehensive overview of all published studies on the topic (until the end of 2022) in order to create a starting point for other researchers in this area, as is common for Systematic Evidence Maps (https://systematicreviewsjournal.biomedcentral.com/articles/10.1186/s13643-016-0204-x). We worded the research question both in the protocol and in the current manuscript as such: "The research goals were '(1) to provide a comprehensive overview over the current state in published empirical research that focuses on self-efficacy concerning exercise behaviour, (2) to identify the currently existing subtopics of self-efficacy in that context, (3) to compile the quantity of research that has been done on the different subtopics and (4) to enable other researchers to systematically justify and derive research gaps in their specific field' ".

Since adding effects would constitute a major change to the protocol and a significantly wider scope, we could not realistically add this to the systematic evidence map.

To manage future readers' expectations, we decided to add this point to the limitations section, which now reads:

A limitation of this study is that it does not offer an in-depth analysis of the research topic, thus excluding outcomes and strength of evidence.

Below is section-specific feedback:

Introduction:
- The Introduction can be tightened by reducing amount of text devoted to the health benefits of physical activity and exercise and more quickly moving to the focus on exercise self-efficacy.

ANSWER: Thank you for raising this point. We have tightened the introduction following your advice. The new section reads as follows:

An active lifestyle is equally important for the secondary prevention of numerous conditions. Following the definition by Frederix et al, secondary prevention aims to stop and/or slow down the progress of established [...] disease, to improve functional capacity, to restore quality of life and to

reduce the risk of disease recurrence" [6], there is plentiful evidence for positive effects of exercise for numerous conditions: For stroke, inactivity is a lifestyle risk factor [7]. After a stroke, exercise can improve cardiovascular fitness, walking ability and upper-body muscle strength in patients, and help with post-stroke fatigue [8]. Obese people can benefit from exercise contributing to weight loss, and from an improved cardiorespiratory fitness [9], which reduces mortality even when controlling for BMI [10, 11]. An active lifestyle can reduce the incidence and improve etiopathology of heart disease, hypertension, type II diabetes and osteoporosis [12–16]. For a number of cancer types, there is evidence on physical activity reducing not only incidence, but also mortality if the cancer already occurred [17]. For depressive symptoms, recreational physical activity has been shown to be an independent predictive factor [22] that may reduce the risk of symptom occurrence, as well as improve symptoms in a population with diagnosed depression [23, 24]. The same has been shown for anxiety disorders like panic disorder and agoraphobia [25–27], although the effects seem to be limited to aerobic exercise.

- The authors discuss several constructs relevant to exercise behavior (p. 5, lines 27-34). I think the authors would benefit from introducing self-efficacy in the context of existing behavior change theories and prior literature without delineating other constructs related to exercise behavior. The focus of the evidence map is on self-efficacy, so no need to introduce other constructs to the reader. Tell us why you are focused on self-efficacy for exercise.

ANSWER: Thank you for this advice. We have edited this part of the introduction to focus on prior literature and existing health behaviour theories as requested. We deleted the part about other constructs related to exercise behaviour. The new section reads as follows:

Researchers in public health have considered different factors that influence this gap and have found some consistently emerging ones. Self-efficacy is one of "the most important predictors" of exercise behaviour [32]. It is consistently found in empirical research, as shown by systematic reviewing [35] and influential health behaviour models like the theory of planned behaviour [36] or the Health Action Process Approach [37].

- Minor. "Self-efficacy in exercise behavior" should be "self-efficacy for exercise behavior."

ANSWER: If we understand the linguistical nuances correctly, "self-efficacy for exercise behaviour" would only pertain to self-efficacy specifically toward exercise behaviour (excluding e.g. general self-efficacy) while "self-efficacy in exercise behaviour" would include any types of self-efficacy as long as they are in any way connected to exercise behaviour in the respective study. If our understanding is correct, we would like to leave it as-is since we intended to cast a wide net with a sensitive search to ensure a comprehensive selection of studies, as opposed to excluding measures of self-efficacy that were not specific for exercise behaviour.

Results:

- Commonly, systematic evidence map provides a figure of the map of the evidence. The authors do not provide an evidence map. The main limitation is that the authors do not map the evidence of the effects of exercise self-efficacy for key outcomes.

ANSWER: Thank you for this critique. As addressed in the General Comments to the Author above, we agree that effects are of high interest, however out of scope for the current paper.

We map the data points we committed to extracting in the protocol, with visual representations of publications per year, represented age groups and pre-existing conditions, as well as lists for representing type of sports and instruments for measuring self-efficacy.

Discussion:
- Overall, the Discussion sufficiently covers the findings, but is challenged by the lack of relevant gaps and research directions provided by this review. The utility of the evidence map for researchers is limited.

ANSWER: Thank you for raising this point. We reworded part of the discussion to communicate research directions more clearly. Additionally, we included an explicit verbalisation of the research gaps. We hope that this improves the utility of our evidence map for future researchers. The updated part of the discussion reads as follows:

In future research, we propose expanding into this direction as well as updating with new studies published in 2023 and later. A review that assesses the robustness of self-efficacy scales and the effects of heterogeneity of wording (as discussed above) would be relevant. A further research direction pertains to how different target groups understand certain phrasing, namely whether self-efficacy statements are usually interpreted as questions of willpower and commitment, or of external barriers like time constraints. It would also help future research to create a nomothetical network of different aspects of self-efficacy in exercise behaviour, to identify the common denominator(s) and to assess the comparability of these specific aspects. We would also propose closing the research gaps we outlined: For public health considerations, it would be important to identify underrepresented disease groups (as discussed above), to which the variation in the represented disease groups in this systematic evidence map is a good start. Concerning different sports, we would welcome more research on specific sports and their differences, for example contrasting different moderate intensity sports versus vigorous intensity sports according to the WHO classification [5] Also, comparing gender differences (or socioeconomic differences) in certain sports, for example their acceptability, popularity, visibility, which could affect self-efficacy and thus success rates in adapting exercise habits.

Reviewer: 2
Dr. Katrine Rolid, Oslo University Hospital
Comments to the Author:
Thank you for the opportunity to review this interesting paper about self-efficacy in exercise behavior. The paper is well written and will be an informative guide for future researchers in the field.

ANSWER: Thank you for the appreciation of our research topic and for your valuable time in reviewing our manuscript, as well as for the positive feedback.

I have some comments to some of the references which is of older date , especially the papers regarding mental health (references 22- 26). The authors could consider to include references of newer date for example Heissel A, et al. Exercise as medicine for depressive symptoms? A systematic review and meta-analysis with meta-regression British Journal of Sports Medicine 2023;57:1049-1057 , doi:10.1136/bjsports-2022-1062 or Singh B et al . Effectiveness of physical activity interventions for improving depression, anxiety and distress: an overview of systematic reviews. Br J Sports Med 2023;57:1203–9. doi:10.1136/bjsports-2022-106195

ANSWER: Thank you for raising this point and for bringing these interesting and highly relevant papers to our attention. We have included both into our introduction. The part regarding mental health now reads as follows:

For depressive symptoms, recreational physical activity has been shown to be an independent predictive factor [22] that may reduce the risk of symptom occurrence, as well as improve symptoms in a population with diagnosed depression [23, 24]. The same has been shown for anxiety disorders like panic disorder and agoraphobia [25–27], although the effects seem to be limited to aerobic exercise.

VERSION 2 - REVIEW

**Reviewer**	**2**
**Name**	**Rolid, Katrine**
**Affiliation**	**Oslo University Hospital, Dep of Cardiology**
**Date**	**19-Aug-2025**
**COI**	

Thank you for the opportunity to review the revised version of the paper. I have no further comments.
